# Computational Mechanics of Form-Fitting 3D-Printed Lattice-Based Wrist-Hand Orthosis for Motor Neuron Disease

**DOI:** 10.3390/biomedicines11071787

**Published:** 2023-06-22

**Authors:** Silvia Badini, Stefano Regondi, Carmen Lammi, Carlotta Bollati, Giordana Donvito, Raffaele Pugliese

**Affiliations:** 1 Nemolab, ASST GOM Niguarda Cà Granda Hospital, 20162 Milan, Italy; silvia.badini@nemolab.it (S.B.); stefano.regondi@nemolab.it (S.R.); giordana.donvito@nemolab.it (G.D.); 2 NEuroMuscular Omnicenter (NEMO), 20162 Milan, Italy; 3 Department of Pharmaceutical Sciences, University of Milan, 20133 Milan, Italy; carmen.lammi@unimi.it (C.L.); carlotta.bollati@unimib.it (C.B.)

**Keywords:** additive manufacturing, 3D printing, material extrusion, lattice structures, finite element method, poly-ε-caprolactone, motor neuron disease, amyotrophic lateral sclerosis

## Abstract

Motor neuron disease (MND) patients often experience hand-wrist muscle atrophy resulting in severe social consequences and hampering their daily activities. Although hand-wrist orthosis is commonly used to assist weakened muscles, its effectiveness is limited due to the rapid progression of the disease and the need for customization to suit individual patient requirements. To address these challenges, this study investigates the application of three-dimensional (3D) printing technology to design and fabricate two lattice structures inspired by silkworm cocoons, using poly-ε-caprolactone as feedstock material. Finite element method (FEM) analysis is employed to study the mechanical behavior, enabling control over the geometric configuration incorporated into the hand-wrist orthosis. Through tensile displacement and three-point bending simulations, the stress distribution is examined for both lattice geometries. Geometry-1 demonstrates anisotropic behavior, while geometry-2 exhibits no strict directional dependence due to its symmetry and uniform node positioning. Moreover, the biocompatibility of lattices with human skin fibroblasts is investigated, confirming excellent biocompatibility. Lastly, the study involves semi-structured interviews with MND patients to gather feedback and develop prototypes of form-fitting 3D-printed lattice-based hand-wrist orthosis. By utilizing 3D printing technology, this study aims to provide customized orthosis that can effectively support weakened muscles and reposition the hand for individuals with MND.

## 1. Introduction

Motor neuron disease (MND), also known as amyotrophic lateral sclerosis (ALS), is an idiopathic, fatal neurodegenerative disease that affects both upper and lower motor neurons leading to progressive muscle wasting and, ultimately, death [1,2]. As reported by Campbell et al. [3], the average onset is 55 to 60 years old, and the ratio of males to females is 1.5 to 1. Sixty-five percent of ALS patients develop upper body weakness ranging from the proximal weakness of shoulder muscles to distal weakness involving wrist and intrinsic hand muscles [4,5,6]. In ALS patients, hand muscle atrophy often results in impaired finger movement leading to devastating social and psychological consequences that negatively impact their activities of daily living (ADL) [7,8,9]. Since hand weakness interferes with ADL requiring fine motor skills, such as grasping, gripping, and pinching, such patients may have difficulties with doorknobs, buttons, zippers, writing, cutting food, and opening cans and jars. Furthermore, Ivy et al. [10] reported that, with progressive weakness, it is common for patients to have weakness of the wrist extensors leading to wrist drop, which dramatically interferes with grip. 

As reported by Ivy et al. and Tanaka et al., wrist-hand orthosis is generally effective for these disabilities as it can be useful for (1) supporting weak or ineffective muscles and joints, (2) repositioning the hand to a functional position, (3) preventing joint contractures and muscle shortening due to muscle imbalance, and (4) preventing pain associated with the resting position [10,11]. 

Several studies report that a static resting wrist-hand support orthosis may increase the patient’s ability to hold objects and improve overall function [12,13,14,15]. In addition, as reported by Ivy and colleagues [16], a palmar and/or dorsal cock-up orthosis for a flaccid wrist may be beneficial as it allows for greater mobility of the hand and fingers.

However, the application of such orthosis for the management of MND presents certain challenges. Firstly, predicting the functional prognosis of MND can be difficult due to the heterogeneous presentation of symptoms and the rapid progression of the disease. As a result, there is a risk of orthosis becoming ineffective if there is a delay in their implementation. Furthermore, even when orthoses are used, their duration of effectiveness may be limited as the disease progresses and the patient’s daily activities become increasingly impaired. Therefore, early production and use of orthosis are crucial in improving the quality of life for patients with MND. Secondly, the use of orthosis must be flexible to adapt to the stage and condition of the disease. However, physicians often struggle to identify the optimal orthosis for their patients. This emphasizes the need for a multidisciplinary approach in orthotic management. Lastly, commercially available orthotic devices may not fully address the concerns of MND patients. Thus, the customization of orthosis to individual patient needs is essential to improve their effectiveness and, ultimately, the patient’s quality of life (QoL).

In addition, despite recent innovations in commercially available materials for wrist-hand orthoses, they continue to suffer from similar issues as traditional plaster or thermoplastic casts. These issues include stiffness, discomfort, poor ventilation, and difficulty with dressing. To improve patient comfort and compliance, there is a need for the development or use of novel materials that offer greater breathability and flexibility, perhaps biomaterials used in the precision and regenerative medicine sectors [17,18,19,20]. Such materials would allow for increased patient mobility, improve ease of use, and ultimately lead to better patient outcomes. Additionally, new designs should be explored to enhance the aesthetic appeal of the orthosis and reduce the stigma associated with wearing them, further improving patient acceptance and adherence. 

In this scenario, three-dimensional (3D) printing, also called additive manufacturing (AM), has emerged as a highly disruptive technology with the potential to meet the healthcare request for more custom-made devices—including wrist-hand orthosis—both for patients and physicians. This technology offers several advantages over traditional manufacturing methods, including increased design flexibility, shorter lead times, and reduced costs [21,22,23,24,25].

One of the key advantages of 3D printing is the ability to produce personalized orthoses based on patient-specific measurements and anatomical variations [26,27,28]. This personalized approach can lead to better fit, improved comfort, and increased patient satisfaction. Furthermore, as recently reported by Pugliese and Graziosi, 3D printing can also facilitate the production of complex geometries and intricate designs that would be difficult or impossible to achieve with traditional manufacturing methods [20]. Another advantage of 3D printing is the ability to optimize the material properties of the orthotic device. This can be achieved by selecting and computationally testing the mechanical behavior of materials (e.g., stiffness, flexibility, stress resistance) to match the patient’s needs. In addition, 3D printing allows for the fabrication of porous structures that offer improved ventilation, reducing the risk of skin irritation and infection. Given these advantages, 3D printing has the potential to revolutionize the field of orthotics by offering a more personalized and efficient approach to device fabrication. This is particularly important in the case of wrist-hand orthosis for MND, where patient comfort and compliance are critical factors in achieving successful outcomes.

For these reasons, we herein investigate the capability to design lattice geometries for 3D printing custom-made wrist-hand orthoses for MND patients, combining material extrusion (MEX) with the finite element method (FEM) to assess their printability and predict their mechanical behaviors, useful for obtaining form-fitting behavior. We focused our attention on generating and examining two types of lattice geometries (i.e., reticular and triangular as the unit cells) inspired by the silkworm cocoon structures [29]. In addition, paying attention to the available biopolymers already adopted in precision and regenerative medicine, we used poly(ε-caprolactone) (PCL) because of its high printability, soft-like properties, and biocompatibility [30,31,32,33]. Each PCL-based geometry was 3D-printed in order to fine-tune the printing parameters and assess the printability limitation. 

Further, the mechanical behavior of such lattice geometries was studied using FEM, evaluating tensile modulus, compressive modulus, and bending in linear hyperelastic models. The CAD-based FEM method allowed us to predict and understand the mechanical properties of the engineered lattice geometries, thus providing tools for the design and control of the geometric configuration of the lattice structures to be embedded in the hand-wrist orthosis. In addition, we probed the impact of such 3D-printed PCL-based geometries on the viability of human skin fibroblast BJ-5ta, demonstrating their biocompatibility by promoting cell adhesion and growth on the surface of the porous structures.

Lastly, based on the results of the semi-structured interview conducted by an occupational therapist with patients with ALS to assess the functional and structural aspects that wrist-hand orthoses should have to meet their needs, the 3D-printed lattice-based hand-wrist orthosis was designed and produced. The prototypes were then presented to the participants, and they were asked to rate their satisfaction with the device on a five-point Likert scale.

In conclusion, this study, with a bottom-up approach, successfully demonstrated the capability to design and 3D print custom-made hand-wrist orthoses for MND patients using lattice geometries inspired by silkworm cocoon structures. In addition, the FEM analysis revealed that the mechanical properties of these lattice structures are predictable and can be controlled through design parameters, which is important for achieving the form-fitting behavior of hand-wrist orthoses. Furthermore, thanks to the semi-structured interview with ALS patients, we designed and produced the 3D-printed lattice-based hand-wrist orthoses and evaluated their satisfaction through patient feedback. 

This study provides valuable insight into the development of customized orthoses that better meet the needs of individuals with MND. The lattice-based orthoses offer a promising solution for creating form-fitting devices that are both mechanically reliable and biocompatible.

## 2. Materials and Methods

### 2.1. Materials

Poly-ε-Caprolactone (PCL, Mw of 50,000 g × mol^−1^) 46 Shore D with a 1.75 mm diameter (Facilan™ PCL 100, 3D4makers, bp haarlem, The Netherlands) was used as polymer filament without further modifications. All materials were handled with gloved hands and followed standard surface analysis laboratory practices to minimize any possible contamination.

### 2.2. Design of Lattice Geometries

Two different lattice geometrical designs were created using Autodesk Inventor^®^ (Autodesk 2020, McInnis Parkway, San Rafael, CA, USA), simulating the superimposition of different paths during the 3D printing procedure. In order to create the structures to be tested, each pattern was considered as a linear repetition of a 4 mm unit cell size. All designed lattices featured 10 × 10 cell units in the *x*- and *y*-directions, with 2 mm of thickness. The theoretical porosity of the lattice models was obtained by measuring the occupied volume of each lattice, and the designed porosity (***P***) was calculated as follows (Equation (1)) [30]:

(1) $$P=\left(1-\frac{V_{f}}{V_{tot}}\right)\times 100$$

In which *V_f_* is the occupied volume of the lattice, and *V_tot_* is the bulk volume of the model.

### 2.3. Finite Element Method (FEM)

The mechanical behavior of designed lattice geometries was simulated by finite element analysis using 3DEXPERIENCE^®^ (Dassault Systemes, Waltham, MA, USA). To generate FEM models, each CAD model was imported into the FEM software as a continuous unique part. The material properties of PCL were simulated in linear elastic behavior. The elastic modulus and Poisson’s ratio of PCL were 350 MPa and 0.3, respectively, according to the previous report [30]. The elastic modulus was then used in the FE simulation with the assumption of linear elastic deformation and considering the PCL material as isotropic. Ten nodes of quadratic tetrahedral elements with four integration points denoted by C3D10 in Abaqus were used based on the geometry of each FEM model. One of the considerable aspects of FEM analysis is the dependency of its result accuracy on the mesh size since the FEM model with coarse mesh is not representative of the continuous model and leads to deviation from exact results. Thus, a mesh sensitivity analysis was performed by constantly decreasing the mesh size (or increasing the number of elements) to reach mesh size-independent results. After performing this analysis, a suitable mesh size on the FEM models was obtained for the following FEM analysis. The Nlgeom option was set to on in order to allow results with nonlinear behavior. The Nlgeom option was enabled to handle large deformations, which can include significant displacements. By employing the incremental approach with Nlgeom active in dealing with material non-linearity, the model effectively handled the large displacements [34]. An automatic incrementation was selected for the simulation, with a maximum number of increments equal to 100 and an increment size increasing from an initial size of 0.01 up to 0.1 to speed up the process. No particular problems in the iterations were registered. The minimum increment size was left to a default 1.0 × 10^−5^ value. The output requested from the simulations contained stresses, strains, displacement, and force/reaction to allow us to calculate the related elastic and compressive moduli. These variables were calculated for every n number of increments. Then, tensile simulation was performed on the CAD models. The loading face was constrained along the *x*-direction first and along the *y*-direction afterward, while an imposed 1 mm displacement was applied on the *y*- and *x*-direction, respectively (Figure S1A). Then, compression simulation was performed on the CAD models constraining the lower face of the scaffold, denying any possible movement or rotation with respect to the reference system, and 1.5% compressive strain was exerted on the top of the model.

In order to appreciate the elastic behavior of each lattice geometry under bending conditions, a three-point bending simulation was carried out, and the force-displacement diagram obtained for a 10 mm vertical displacement was registered and then compared between models. Two lines were created on each model to simulate the supports of the bending test, and one additional line was created to simulate the loading point. The support distance was fixed equal to 100 mm, and the load was placed at the midspan. Each model created was sized in order to have almost the same value of width to make the results comparable. The complex geometry provided by different lattices results in a different bending behavior depending on the position of the load applied to the models created. Two different types of bending simulation were adopted, the first one considering the support distance parallel to the *x*-axis and the second one with the support distance parallel to the *y*-axis (Figure S1B).

### 2.4. Material Extrusion (MEX) and Printing Parameters

All lattice geometries and wrist-hand orthoses were 3D-printed using the MEX technology through Sharebot 43 (Nibionno, Italy) machine adopting the direct drive extruder suitable for soft materials. The printer has a maximum building volume of 300 × 250 × 200 mm and a layer resolution of 0.2 mm, corresponding to a nozzle with a 0.4 mm diameter. PCL filaments were extruded at a temperature of 160 °C with a bed temperature of 40 °C. Moreover, the following printing parameters were kept constant: extrusion multiplier (1), extrusion width (0.42 mm), retraction distance (1.3 mm), retraction speed (1800 mm/min), fan speed (100%), infill (100%), and printing speed (2500 mm/min) (Table 1). Each lattice and orthosis were modeled using Autodesk Inventor^®^ software, then exported in stereolithography (.stl) format and sliced using Simplify3D^®^ software to be printed. To ensure the adhesion of the structures to the surface of the printing platform, a 3D glue stick (Magigoo™, Swieqi, Malta) was used.

### 2.5. Scanning Electron Microscopy (SEM)

Surface morphology, sagging, improper layer adhesion, and any impurities of 3D-printed lattice geometries were observed using a field emission scanning electron microscopy equipped with an in-lens secondary electron (SE) detector and operating at 5 kV (Zeiss EVO 50). All specimens were sputter coated with gold prior to the examination to ensure better conductivity and prevent the formation of electrostatic charges.

### 2.6. Cell Culture Experiments

Cell condition: BJ-5ta cell line was bought from ATCC (ATCC from LGC Standards, Milan, Italy), cultured in DMEM high glucose and Medium 199 (4:1 ratio) with stable L-glutamine supplemented with 10% FBS, 100 U/mL penicillin, 100 µg/mL streptomycin and incubated at 37 °C under a 5% CO_2_ atmosphere. In the cell culture experiments, 3D-printed lattices were printed and then autoclaved before the cell experiments. Afterward, 3D-printed lattices were transferred to 24-well plates and incubated with 500 µL of complete medium for 2 h at RT.

Cell viability assessment: a total of 2.5 × 10^5^ BJ-5ta cells/well were seeded in 24-well plates in the absence (control cells) and in the presence of 3D-printed lattices in 500 µL of complete growth media for 48 h at 37 °C under 5% CO_2_ atmosphere. Subsequently, the medium was discarded, and 500 µL/well of 3-(4,5-dimethylthiazol-2-yl)-2,5-diphenyltetrazolium bromide (MTT) filtered solution was added. After 2 h of incubation at 37 °C under a 5% CO_2_ atmosphere, a 0.5 mg/mL solution was aspirated, and 500 µL/well of the lysis buffer (8 mM HCl + 0.5% NP-40 in DMSO) was added. After 30 min of slow shaking, the absorbance of the solutions was read at 575 nm on the Synergy H1 fluorescence plate reader (Biotek, Bad Friedrichshall, Germany). Each experiment was performed in triplicate.

### 2.7. Statistical Analysis

Statistical analysis was carried out by one-way ANOVA (GraphPad Prism 9) followed by Tukey’s multiple comparisons test. Values were expressed as means ± SEM; *p*-values < 0.05 were considered to be significant.

### 2.8. User Satisfaction Questionnaire

Ten individuals diagnosed with definite or presumable ALS, based on the El Escorial Criteria [35], were recruited for the semi-structured interview. All participants had prior experience with a wrist-hand orthosis. Prior to the semi-structured interview, all patients provided informed consent. The semi-structured interview was conducted by an occupational therapist and was specifically designed to assess the functional and structural aspects that wrist-hand orthosis should have to meet the needs of individuals with MND. The interview evaluated several key aspects, including size, weight, material, breathability, shape, thickness, ease of use, and customization. Based on the results of the semi-structured interview, 3D-printed orthoses were designed and produced. The prototypes were then presented to the participants, and they were asked to rate their satisfaction with the device on a 5-point Likert scale using Quebec User Evaluation of Satisfaction with Assistive Technology (QUEST, V2.0). A score of 5 indicated that the participant was “very satisfied”, while a score of 1 indicated that they were “not satisfied at all”.

## 3. Results and Discussion

### 3.1. Design and Prototyping of Lattice-Based Geometries

Bio-inspired structures that imitate natural designs have gained increasing attention in recent years [36,37,38]. The development and exploration of novel bio-inspired structures are motivated by the desire for geometries that possess superior mechanical properties, such as high strength, flexibility, and breathability, without compromising on efficiency and the ability to remain lightweight [39].

**Figure 1:**
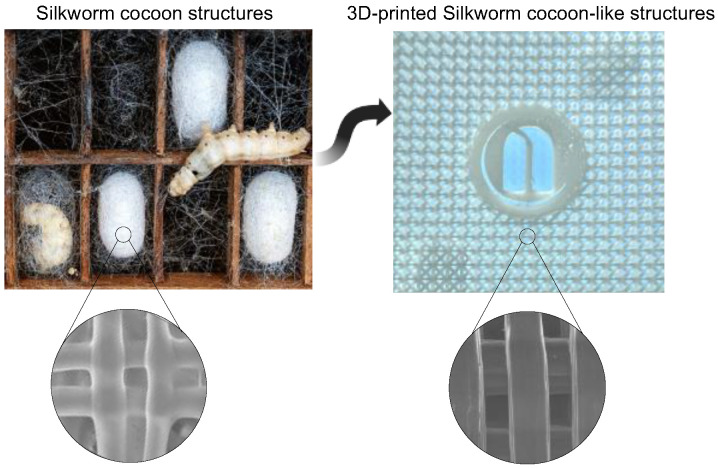
A 3D-printed lattice structure inspired by the architecture of silkworm cocoons. In the lower part of the figure, a scanning electron microscopy (SEM) enlargement is presented, showing a comparison between the structure of silkworm cocoons and the 3D-printed bioinspired lattice.

Drawing inspiration from nature, our objective was to create a lattice geometry that could satisfy these criteria as well as offer additional benefits, such as sustainability and bio-compatibility. In this way, it might be possible to create structures that require fewer resources and have a smaller environmental footprint, as well as structures that are bio-compatible, reducing the risk of rejection or adverse reactions when used in biomedical applications. Hence, we drew inspiration from the architecture of silkworm cocoons to design and 3D print a lattice with exceptional mechanical properties [40] (Figure 1). Silkworm cocoons are a natural structure that has evolved over millions of years and possess unique features that make them resilient and adaptable.

Their layered structure provides structural support and porosity, enabling gas exchange and respiration. Despite their strength and resilience, they are remarkably lightweight.

**Figure 2:**
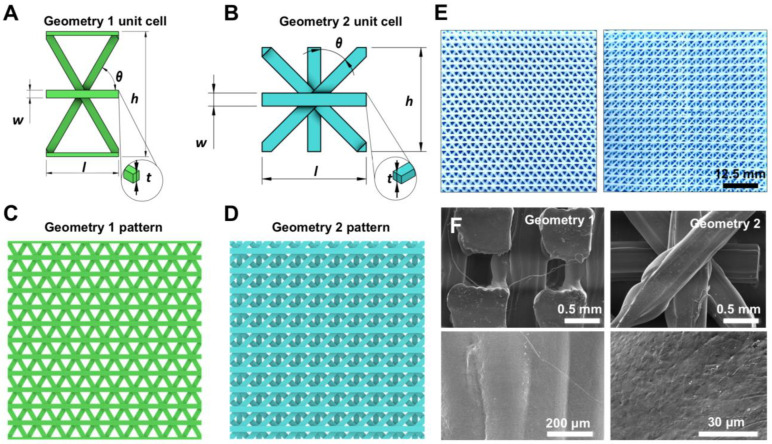
(**A**,**B**) The two different unit cell size designs and their main geometrical parameters (namely l, h, w, t, and θ). (**C**,**D**) The two different lattice patterns investigated featuring 10 × 10 cell units. (**E**) Three-dimensionally-printed 10 × 10 lattice pattern using PCL material. (**F**) SEM images of the PCL-based lattice structures.

We created two unit cell geometries (Figure 2A,B) by analyzing the natural Silkworm cocoons structures and incorporating specific geometrical parameters (l, h, w, t, and θ) listed in Table 2. Once designed as a surface, the unit cells were replicated 10 × 10 in order to achieve two final complex patterns (Figure 2C,D) that can be 3D-printed with optimal mechanical properties.

The unit cells were designed to replicate the deposition mechanism of the MEX process for the PCL filament, and each unit cell was generated to distribute loads evenly and ensure stability. Therefore, the geometries are arranged, layer-by-layer, by a network of struts and nodes that are in charge of ensuring the structure’s stability and distributing loads evenly.

Specifically, the value of the thickness (t) is equal to the double layer height, which corresponds to the printing nozzle diameter (0.4 mm), while the width (w) value corresponds to the first layer width (0.5 mm), which is higher than the nominal extrusion width due to necessity of adherence of the printed part to the heated platform. Both patterns were designed to allow the MEX machine to create suspended layers without additional support. Printing parameters, listed in Table 1, were selected to control the flow and correct filament deposition. A retraction distance of 1.3 mm and a vertical retraction lift of 0.6 mm were selected as parameters of major significance to avoid lumps and strands. The lattice-based geometries were 3D-printed successfully (Figure 2E) with high accuracy and quality, resulting in smear-free patterns. Additionally, geometry 1 and geometry 2 enclose 80% and 78% of pore fraction, respectively, thus providing appropriate breathability and lightweight (Figure S2).

Our findings were confirmed by scanning electron microscopy (SEM) analysis (Figure 2F), which showed that none of the 3D-printed lattices exhibited defects such as sagging, improper layer adhesion in node locations, or intrusive porosities. At higher magnifications, the smooth surface of the material extruded during the printing was clearly visible. These results further demonstrate the high accuracy and quality of our lattice-based structures.

### 3.2. In Vitro Biocompatibility of Lattice-Based Geometries

To assess the biocompatibility of 3D-printed lattice structures made of PCL for potential use in wrist-hand orthoses that come into contact with human skin, we conducted in vitro experiments using human skin fibroblasts. Furthermore, the in vitro test was useful to determine whether the standard biocompatibility of PCL material [41,42,43], which makes it suitable for biomedical applications, is affected by the 3D printing method.

To evaluate the capability of the 3D-printed PCL-based lattice structures to support cell adhesion and proliferation, we seeded a total of 2.5 × 10^5^ BJ-5ta cells/well directly on the top surface of the 3D-printed structures. The cells were cultured for 1 and 3 days in vitro (DIV), and their morphology was examined after 3 DIV. In addition, we assessed cell viability via MTT assay.

**Figure 3:**
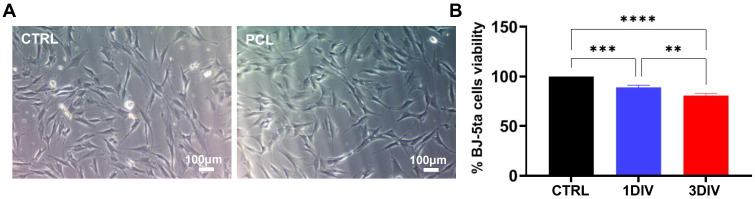
(**A**) Morphology and (**B**) viability of BJ-5ta cells cultured for 1 and 3 DIV. No significant difference in cell morphology was detected between cells cultured on 3D-printed PCL lattices and untreated plastic wells. The MTT assay assessing cell viability showed revealed no cytotoxic effects after both 1 and 3 DIV on 3D-printed PCL lattices. ** *p* < 0.01; *** *p* < 0.001; **** *p* < 0.0001.

As shown in Figure 3A, the BJ-5ta cells cultured over 3D-printed PCL lattices exhibited spread, fusiform, and branched cellular shapes without significant morphological changes compared to cells cultured over untreated plastic wells (negative control, named CTRL). Furthermore, the MTT assay revealed no cytotoxic effects after both 1 and 3 DIV, indicating good biocompatibility of the 3D-printed PCL-based lattice structures (Figure 3B).

Overall, our findings suggested that the 3D-printed PCL-based lattice structures are suitable for use in hand-wrist orthoses that come into contact with human skin. However, we are aware that more studies are needed to assess the long-term effects of these structures on the skin and to investigate their potential for clinical applications.

### 3.3. Finite Element Analysis

**Figure 4:**
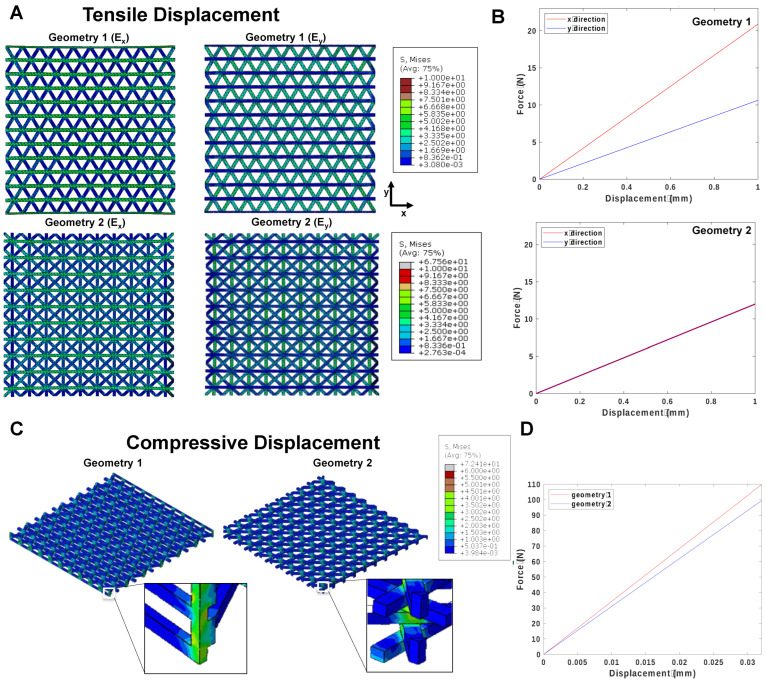
Mechanical behavior analysis of two designed geometries under tensile and compression. (**A**) Distribution of von Mises stress under tensile displacement for both designed geometries (the stress value increases from blue to red colors). (**B**) Force variation between *x*- and *y*-direction in geometry 1 and 2 under tensile load. (**C**) The von Mises stress–strain distribution in the simulated compression test by FEM for both geometries (the magnification shows that geometry 1 has thicker load nodes compared to geometry 2). (**D**) Comparison of the amount of force after completion of analysis for geometry 1 and geometry 2 under compression.

In order to conduct linear FEM analysis, the elastic modulus of the PCL filament was assumed to be 350 MPa, and Poisson’s ratio was set to 0.3 (see Materials and Methods for more details). Using this modulus, the mechanical behavior of the two designed geometries under tensile and compression was simulated and analyzed (Figure 4).

The distribution of von Mises stress under tensile displacement for both geometries is depicted in Figure 4A. Interestingly, we observed a variation of load vs displacement between the *x*-direction and *y*-direction in geometry 1 (Figure 4B), indicating that the horizontal layers interconnected with nodes (visible in E_x_) play a significant role in its resistance to a tensile load. In contrast, the maximum stress amount for geometry 2 was evenly distributed between the *x*- and *y*-direction due to the uniform positioning of the nodes. After the FEM simulation, the amount of force and equivalent tensile modulus was calculated for both geometries. The tensile modulus of geometry 1 was found to be 12.56 MPa and 6.92 MPa for the *x*-direction and *y*-direction, respectively. On the other hand, the tensile modulus of geometry 2 was 7.50 MPa for both directions.

Next, the mechanical behavior under compression was simulated for both geometries, and the distribution of von Mises stress is displayed in Figure 4C. We observed a slight increase in the amount of force after the completion of analysis for geometry 1 compared to geometry 2 (Figure 4D), which we attributed to the existence of thicker load nodes (magnified in Figure 4C), in agreement with previously reported results on PCL lattice scaffolds [30]. Accordingly, the elastic modulus in compression was calculated by the ratio of compressive stress to compressive strain and found to be 7.48 MPa and 5.52 MPa for geometry 1 and geometry 2, respectively.

**Figure 5:**
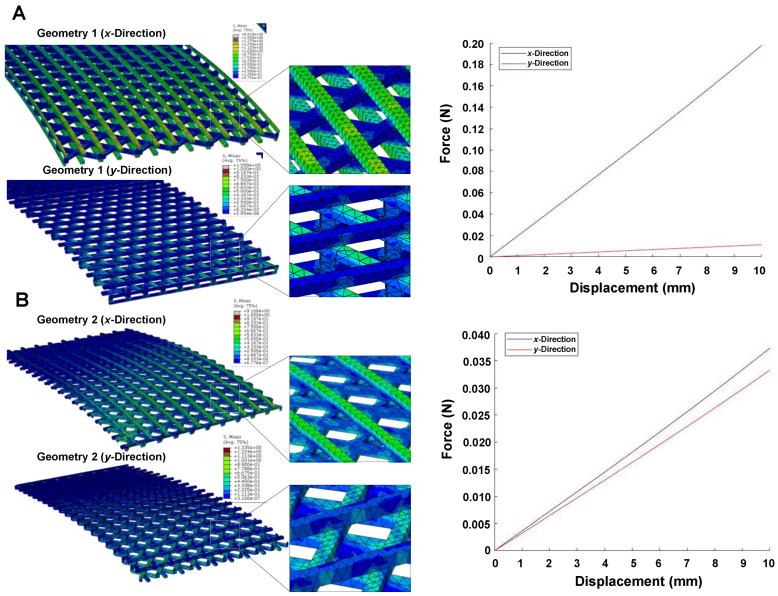
(**A**) Von Mises stress distribution and load vs displacement curves in the *x*-direction and *y*-direction for geometry 1, showing highly distributed stress values predominantly in the *x*-direction. (**B**) Von-Mises stress distribution and related load vs displacement curves for geometry 2, exhibiting no strict directional dependence. Slight variations are observed in the curves between the *x*- and *y*-directions.

In addition, a three-point bending simulation of each lattice geometry was carried out (Figure 5). Figure 5A,B shows FEM prediction for von Mises stress distribution in both PCL-based geometries during the bend test. We observed that the stress values for geometry 1 are highly distributed and spread in the *x*-direction (Figure 5A). This finding was consistent with the tensile displacement data, indicating that the load was higher in the *x*-direction compared to the *y*-direction, thus confirming the anisotropy feature of such geometry.

In contrast, the von Mises stress distribution for geometry 2 exhibited no strict directional dependence. This can be attributed to the symmetry of the geometry and the uniform positioning of the nodes (Figure 5B). However, we did notice slight variations in the load vs displacement curves between the *x*- and *y*-directions for this geometry.

Overall, the FEM simulations of the three-point bending behavior provided valuable insights into the stress distribution, deformation patterns, and potential failure points for the lattice geometries intended for the wrist-hand orthosis. This information is crucial for designers and engineers as it helps optimize the structural integrity of the orthosis. By considering these findings, design modifications, such as incorporating structural reinforcements or optimizing the shape, can be implemented to enhance load distribution and improve wearer comfort.

Ultimately, in the context of this study, this bending information can aid in choosing the most suitable geometry that ensures optimal form fitting when the orthosis is worn.

### 3.4. Possible Application of 3D-Printed Wrist-Hand Orthosis for Motor Neuron Diseases

Lastly, the functional and structural aspects necessary for 3D-printing wrist-hand orthosis to meet the needs of individuals with MND were explored through semi-structured interviews (see Materials and Methods). Ten participants diagnosed with definite or presumed ALS were recruited, all of whom had prior experience with a hand-wrist orthosis. The interviews focused on evaluating various aspects such as weight, material, breathability, shape, thickness, ease of use, and customization.

**Figure 6:**
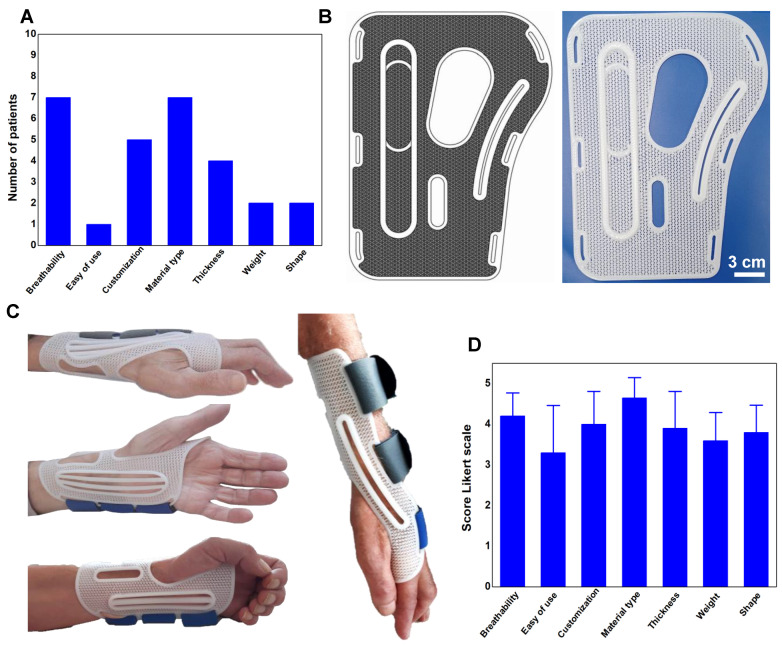
(**A**) Key aspects that 3D-printed wrist-hand orthosis must possess to meet the needs of individuals with MND. (**B**) CAD-design and 3D printing of wrist-hand orthosis embedded with lattice geometry 1 for MND. (**C**) The wearability of 3D-printed lattice-based orthosis highlighting the form-fitting behavior. (**D**) The satisfaction ratings of participants on the prototypes of the 3D-printed lattice-based hand-wrist orthosis.

Figure 6A illustrates the key considerations that emerged from the interviews. Material type and breathability were rated as important aspects by 7 out of 10 patients, while customization according to individual patient needs was highlighted by 5 out of 10 patients. Other notable aspects included thickness (rated by 4 out of 10 patients), weight and shape (rated by 2 out of 10 patients), and ease of use (rated by 1 out of 10 patients). Based on these results, 3D-printing wrist-hand orthoses were designed and manufactured.

Considering the FEM simulations, geometry 1 exhibited superior results in terms of pores fraction, elastic modulus, and three-point bending behavior. Thus, this geometry was selected for designing the wrist-hand orthosis. The anisotropic feature observed during the three-point bending simulation proved particularly valuable. By aligning the stiffer *x*-direction parallel to the arm and the softer *y*-direction perpendicular to the arm, optimal adaptation to the shape of the limb was ensured, providing a form-fitting experience. This property is especially advantageous as it eliminates the need for thermoforming the polymer, which is often required for other types of orthotics available in the market. The PCL-based orthosis immediately conforms to the limb’s shape upon wearing and returns to its original shape upon removal.

With inspiration from silkworm cocoon structures, novel wrist-hand orthoses embedded with lattice geometries were designed and engineered for MND (Figure 6B).

The production process consisted of two phases: gathering measurements of the anatomical region of interest to generate the initial orthosis representation, followed by the creation of orthosis edges and reinforced portions through extrusion. Subsequently, lattice geometry 1 was repetitively applied and cut to match the inner boundary of the orthosis. The combined boundary and lattice pattern were exported in stereolithography (STL) format and sliced using Simplify3D^®^ software.

By utilizing the selected printing parameters, stable and self-supporting structures have been successfully achieved. Further, such printing parameters resulted in the final 3D-printed orthosis that met the desired form-fitting characteristics, with a thickness of 4 mm, weight of 20 g, and porosity of over 80% (Figure 6C). Furthermore, the optimization of printing parameters has enabled the orthosis to be fabricated using the MEX method within just 40 min. This achievement addresses one of the key challenges associated with the utilization of such orthosis in MND, namely reducing the time gap between the planning phase and the actual use of the orthosis. This time reduction is crucial to ensure that the orthosis is not perceived as ineffective for the patient, thus enhancing its practicality and timely delivery for improved patient outcomes.

Finally, the participants were presented with prototypes of the 3D-printed lattice-based hand-wrist orthosis, and their satisfaction with the device was assessed using a five-point Likert scale. A score of 5 denoted “very satisfied”, while a score of 1 indicated “not satisfied at all”. Figure 6D shows the results, revealing notable ratings in several aspects. The customization potential of the orthosis received a score of 4, indicating high satisfaction among the participants. Similarly, the type of biomaterial employed and the breathability achieved impressive scores of 4.65 and 4.1, respectively. From a structural design perspective, the size, shape, and thickness parameters attained scores of 3.6, 3.8, and 4, respectively. These findings shed light on the participants’ perspectives and emphasize the importance of customization, biomaterial selection, and breathability in optimizing orthosis satisfaction.

Given these promising data, future clinical studies should explore the effectiveness of 3D-printed lattice-based orthosis in addressing the specific needs of individuals with MND conditions. By experimentally investigating their impact on muscle and joint functionality, range of motion, pain reduction, and overall patient satisfaction, these studies can provide valuable insights and contribute to the management of 3D-printed orthotics in neuromuscular and neurodegenerative diseases.

## 4. Conclusions

In conclusion, this study highlights the potential of computational mechanic simulations and 3D printing technology in the design and fabrication of personalized wrist-hand orthosis for patients with MND. The use of lattice geometries inspired by silkworm cocoon structures offers an interesting approach to creating form-fitting orthoses that address the specific needs of MND patients.

The combination of MEX methods with FEM allowed for the assessment of the printability and mechanical behavior of the lattice geometries, demonstrating that their mechanical features could be predicted and controlled through design parameters, enabling the achievement of form-fitting behavior in hand-wrist orthoses. The simulated tensile modulus of geometry 1 was determined to be 12.56 MPa and 6.92 MPa along the *x*- and *y*-directions, respectively. In contrast, geometry 2 exhibited a tensile modulus of 7.5 MPa in both directions. Compression FEM analysis revealed elastic moduli of 7.48 MPa for geometry 1 and 5.52 MPa for geometry 2. Additionally, the three-point bending simulations further confirmed the anisotropic behavior observed in the previous tensile displacement results for geometry 1. This anisotropic characteristic is highly desirable for the effectiveness of wrist-hand orthoses.

Moreover, the use of PCL as a biocompatible material for 3D printing proved to be suitable for the fabrication of lattice-based orthoses. Indeed, the MTT assay of BJ-5ta cells revealed no cytotoxic effects after both 1 and 3 DIV, indicating good biocompatibility of the 3D-printed PCL-based lattice structures.

The porous structures created by 3D printing with PCL address issues related to discomfort, poor ventilation, and potential skin irritation associated with traditional orthotic devices.

The study also considered the perspectives of ALS patients through a semi-structured interview. This “patient-centered” approach allowed for the identification of functional and structural aspects that the hand-wrist orthosis should incorporate to meet the specific needs of ALS patients. Significantly, 70% of the patients expressed the importance of material type and breathability as crucial factors, while 50% of the respondents emphasized the need for customization to cater to their individual requirements. Furthermore, patients expressed a strong interest in minimal thickness, aesthetically pleasing shapes, lightweight design, and ease of use. These valuable insights from the patients provided important considerations for optimizing the wrist-hand orthosis design.

Overall, this study demonstrates the feasibility and potential benefits of utilizing 3D printing technology, bio-polymer, and lattice-based designs in the development of custom-made hand-wrist orthoses for MND patients. The ability to create personalized orthotic devices with improved form-fitting, comfort, and mechanical reliability has the potential to enhance the quality of life for individuals with MND. Future research should focus on further refining the design and fabrication processes, exploring additional biomaterial options, and conducting clinical evaluations to assess the impact on muscle and joint functionality, range of motion, pain reduction, and overall patient satisfaction.

## Figures and Tables

**Table 1:** Printing parameters for MEX of lattice-based geometries made of PCL.

*PCL Lattices’ Printing Parameters*	
Extrusion Multiplier (−)	1
Layer height (mm)	0.2
Extrusion width (mm)	0.42
First layer height (%)	150
First layer width (%)	120
Extrusion Temperature (°C)	160
Platform Temperature (°C)	40
Fan speed (%)	100
Printing speed (mm/min)	2500
Outline underspeed (%)	70
Outline overlap (%)	10
Retraction distance (mm)	1.3
Retraction vertical lift (mm)	0.6
Wipe distance (mm)	0
Coasting distance (mm)	0
Infill (%)	100

**Table 2:** Geometrical parameters of the designed lattice geometries according to Figure 2A,B.

Geometry	Geometrical Parameters				
	θ	l	h	w	t
	[°]	[mm]			
1	60	4.62	8	0.5	0.4
2	45	4	4	0.5	0.4

## Data Availability

Data is contained within the article or supplementary material.

## Supplementary Materials

The following supporting information can be downloaded at: https://www.mdpi.com/article/10.3390/biomedicines11071787/s1, Figure S1: FEM details of tensile and three-point bending simulations; Figure S2: Porosity of lattice-based geometry 1 and geometry 2.
