# Supplementary material for: Computational Mechanics of Form-Fitting 3D-Printed Lattice-Based Wrist-Hand Orthosis for Motor Neuron Disease
Source: Biomedicines. 2023 Jun 22;11(7):1787. doi: 10.3390/biomedicines11071787 (PMC10376028; doi:10.3390/biomedicines11071787)
Supplement: Supplementary file 1 [file biomedicines-11-01787-s001.zip › biomedicines-2436242-supplementary.pdf]

biomedicines

Supplementary Information

Computational Mechanics of Form-Fitting 3D-Printed Lattice-Based Hand-Wrist Orthosis for Motor Neuron Diseases

Silvia Badini [1,†], Stefano Regondi [1,2], Carmen Lammi [3], Carlotta Bollati [3], Giordana Donvito [1] and Raffaele Pugliese [1,*,†]

[1] Nemolab, ASST GOM Niguarda Cà Granda Hospital, Milan, 20162 Italy; silvia.badini@nemolab.it (S.B.); stefano.regondi@nemolab.it (S.R.); giordana.donvito@nemolab.it (G.D.)
[2] NEuroMuscular Omnicenter (NEMO), Milan 20162, Italy
[3] Department of Pharmaceutical Sciences, University of Milan, Milan, 20133 Italy; carmen.lammi@unimi.it (C.L.); carlotta.bollati@unimib.it (C.B.)
* Correspondence: raffaele.pugliese@nemolab.it
† These authors contributed equally to this work.

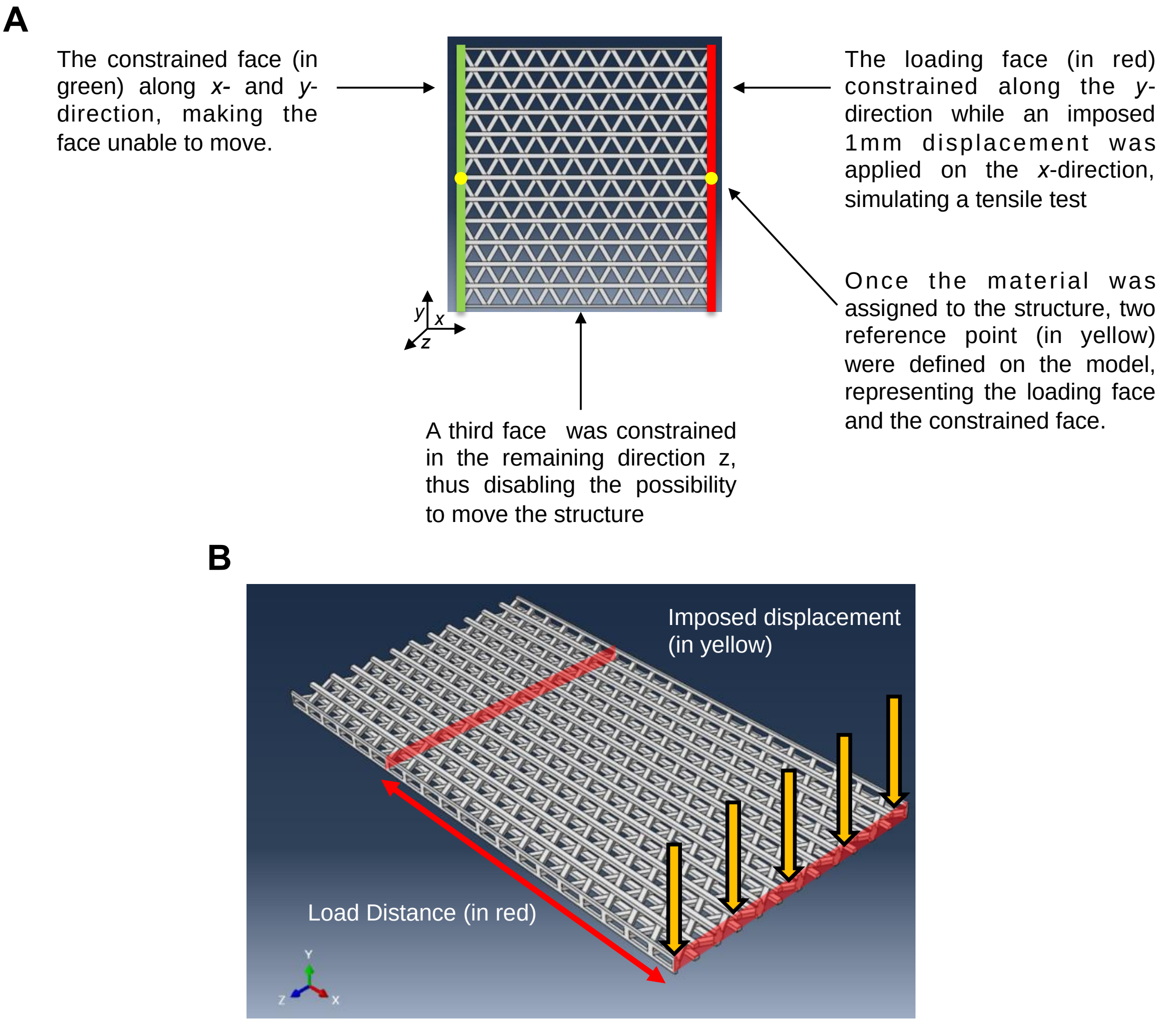

Figure S1. Details of FEM procedures for tensile and three point bending simulations. (A) Tensile test, and (B) three-point-bending simulation.

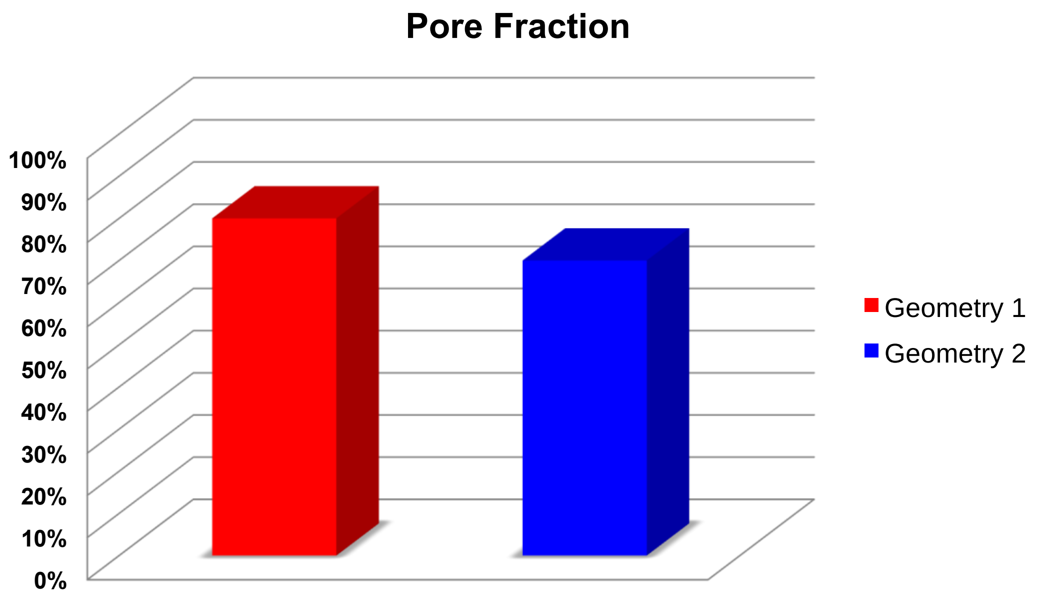

Figure S2. Designed porosity calculation obtained with Equation (1).
